# Long-term *in vitro* degradation behaviour of Fe and Fe/Mg_2_Si composites for biodegradable implant applications

**DOI:** 10.1039/c8ra00404h

**Published:** 2018-03-06

**Authors:** M. Sikora-Jasinska, P. Chevallier, S. Turgeon, C. Paternoster, E. Mostaed, M. Vedani, D. Mantovani

**Affiliations:** Lab. for Biomaterials & Bioengineering (CRC-I), Dept. Min-Met-Materials Engineering & Research Center CHU de Québec, Laval University Pouliot Building 1745G Québec City Canada Diego.Mantovani@gmn.ulaval.ca http://www.lbb.ulaval.ca +1-418-656-2131 ext. 6270; Department of Mechanical Engineering, Politecnico di Milano Milan Italy

## Abstract

The major drawback of Fe-based materials for biodegradable implant applications is their slow degradation rate. Addition of second phase particles into the Fe matrix can increase the degradation rate at the beginning of the corrosion process. However, so far, there is neither quantitative data on *in vitro* degradation nor direct experimental evidence for long-term dissolution of Fe-based biodegradable composites. Here, a series of immersion tests at different exposure intervals (20, 50 and 100 days) to modified Hanks' solution were performed to study the degradation behavior of Fe and Fe/Mg_2_Si composites prepared by different powder metallurgy techniques. The results revealed the role of Mg_2_Si in the composition and stability of the protective films formed during the static corrosion experiments. Fe/Mg_2_Si composites showed higher degradation rates than those of pure Fe at all stages of immersion. Degradation rates at distinct exposure intervals strongly depended on the composition and stability of formed oxide, hydroxide, carbonate and phosphate protective films on the degraded surfaces. The release of Fe ions into the solution at later stages of the experiment was limited due to the barrier effect of the insoluble deposit. This fundamental study provided a basis for the processes of protective film formation in modified Hanks' solution, which enables a detailed identification of its characteristic features.

## Abstract 2

This fundamental study provides a basis for the processes of protective film formation on degradable Fe-based biomaterials.

## Graphical abstract

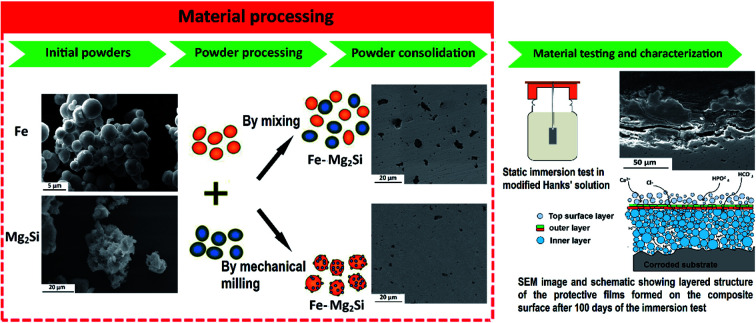

## 1. Introduction

Over the last two decades, many studies on the development of Fe-based degradable implants have been performed.^1–5^ From a mechanical point of view, Fe and its alloys and composites are very attractive for cardiovascular stent applications, due to a good combination of high ultimate tensile strength and acceptable ductility.^3^ Such implants should degrade at an appropriate degradation rate and disappear entirely after total repair of the surrounding tissues. Unfortunately, challenges related to the extremely slow degradation rate of currently available Fe-based materials remain unsolved.^6^

Recently, researchers have focused on the increase in the degradation rate of Fe-based biodegradable metals (BMs) by modifying their chemical composition, microstructure and surface property.^7^ It was proved, that the presence of phases with a difference in corrosion potential from that of the Fe matrix can increase the degradation rate by promoting micro-galvanic corrosion.^8^ The amount, distribution and size of the reinforcement phase might alter the corrosion properties of Fe-based metal matrix composites (MMCs).^5^

To date, corrosion studies have been focused mainly on the calculation of the degradation rates.^9–14^ A detailed investigation on the corrosion initiation, its progression, formation, development, and growth of corrosion products and their precise identification still remains largely unexplored. In most cases, an addition of reinforcement or alloying element could increase the degradation rate of Fe during initial stages of static immersion tests.^4,14–17^ However, the effect of long-term exposure to the physiological solution on the degradation of Fe-based alloys remains poorly understood. More importantly, the lack of data on the formation and composition of the degradation products over time unable considerations on their biosafety. Degradation precipitates generated by the progressively dissolving metal may disturb the physiological equilibrium, thus they should be identified and evaluated. Further, it is obvious that degradation product formation generates volume increase. It can limit material application especially in terms of mechanical properties, causing corrosion induced-failure.^18^ However, the effect of expanding volume of degradation products over time seems to be dismissed in the literature. The latest efforts have brought the development of several testing strategies, that predict the evolution of Fe corrosion and release.^19–21^ A variety of parameters affect the Fe ion release and there is no agreement regarding the quantitative extent that each factor contributes. As a result, a direct comparison between the reported in the literature degradation rates for Fe and Fe-based BMs remains impossible. Although each model might be beneficial for simulating certain mechanisms Fe dissolution, thus far, no single model has been developed, providing a comprehensive depiction of Fe release phenomena.

Previously, the corrosion initiation mechanism for Fe and Fe/Mg_2_Si composites was discussed in detail.^5^ Here, the research is focused on their long-term degradation behavior. A series of immersion tests at different exposure intervals (20, 50 and 100 days) was performed. This fundamental study provides a basis for the sequence of protective film formation in physiological media. Further, the influence of the Mg_2_Si addition on the corrosion of Fe and corresponding mechanism of degradation is explored. Samples' surfaces after immersion at different exposure intervals were thoroughly characterized by X-ray diffraction (XRD). As shown in the previous study,^5^ XRD is not suitable for the detection of the phosphate-based products at shorter exposures to the corrosive environment. Consequently, Fourier transform infrared spectroscopy (FTIR) was used as a complementary characterization method, which is in contrast very sensitive to even nanocrystalline or amorphous phosphate-based degradation products. The combined use of XRD and FTIR allows identifying all the solid phases forming protective films on the top of investigated biomaterials. In this work, details about the degradation behavior at long-term exposure times to the physiological environment are highlighted adding a crucial knowledge on the degradation mechanism of degradable implant materials.

## 2. Materials and methods

### 2.1. Synthesis of composites

Fe and Fe/Mg_2_Si composites were fabricated *via* powder metallurgy process as described in ref. 5. Table 1 summarizes the detailed description of the investigated powder conditions and the corresponding denotation of the investigated specimens.

**Table 1:** Samples investigated in the present study and their denotation

Sample denotation	Composition		Starting powders	Powder preparation	Sample name after *X* days (*X* = 20, 50 or 100) of immersion in modified Hanks' solution		
					20	50	100
PF	Fe	100 wt%	As received		PF20	PF50	PF100
MX	Fe	99 wt%	As received	24 h mixed	MX20	MX50	MX100
	Mg_2_Si	1 wt%	32 h mechanically milled				
MM	Fe	99 wt%	As received	24 h mechanically milled	MM20	MM50	MM100
	Mg_2_Si	1 wt%	32 h mechanically milled				

PF samples were made only of pure Fe powders (99.9% purity supplied by Alfa Aesar) and used as reference materials (Table 1). In case of composites, corresponding to MX and MM conditions, samples were composed by Fe and 1% wt Mg_2_Si powders (99.5% purity, supplied by Sigma Aldrich). Beforehand, the as-received, coarse Mg_2_Si powders were mechanically milled. For MX condition, the powders were mixed in a horizontal cylindrical mixer for 24 hours, while for MM conditions, the powders were mechanically milled in a planetary ball mill (PM 400 Retsch^®^), in an argon atmosphere for 24 hours with a milling speed of 240 rpm. Afterwards, powders were compacted by multi-step hot rolling to a total nominal reduction in thickness of 60%. Before each rolling step, samples were preheated to 700 °C in a resistance furnace for 20 min.

### 2.2. Metallographic examination

Samples for microstructural observation were cut, ground and polished according to the standard metallographic procedure. A FEI Quanta 250 scanning electron microscopy (SEM) with a tungsten filament equipped with an electron dispersive X-ray spectrometry (EDS) was used to investigate the microstructure and morphology of the samples and to analyze the chemical composition of the phases with an acceleration voltage in the range of 10–30 kV. In order to evaluate the reinforcement size distribution, SEM micrographs were analyzed using Microstructure Characterizer software. Feret's equivalent diameter was chosen to estimate the size of the reinforcement. Diffraction patterns of as-received and degraded sample surfaces and degradation products were obtained by X-ray diffractometry (XRD). The Siemens D5000 diffractometer with a curved graphite crystal monochromator operated with a Cu anode (*λ*_Cu Kα_ = 1.54184 nm), an acceleration voltage of 40 kV and a current of 30 mA.

### 2.3. Degradation behavior

The degradation behavior of Fe and Fe/Mg_2_Si composites was studied in Hanks' modified solution. The solution composition and its preparation are reported by Lévesque *et al.*^22^ The solution pH was adjusted to 7.4, by the addition of 1 M HCl or 1 M NaOH, where appropriate. Prior to corrosion tests, the samples surfaces were polished according to the standard metallographic procedure. Static immersion tests were performed to investigate the long-term degradation behavior of the Fe and Fe/Mg_2_Si composites, according to ASTM G31-72 standards. 18 mm × 9 mm × 1 mm samples were immersed for 20, 50 and 100 days in 80 mL of Hanks' modified solution.^22^ The containers were stored in a controlled environment (*T* = 37 ± 1 °C), with a CO_2_ atmosphere content of 5 vol% and a relative humidity of 90%. The solution was changed every 10 days. Subsequently, the samples were washed with 70% ethanol in an ultrasonic bath for 5 minutes in order to remove the degradation products on the surface before measuring the final weight. The corrosion rates of the composites were calculated using the weight loss method according to the following equation:1
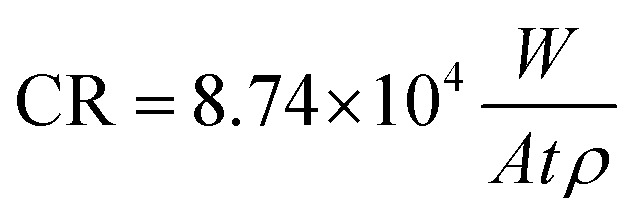
in this equation, CR is the corrosion rate (mm per year), *W* is the weight loss (g), *A* is the exposed area (cm^2^), *t* is exposure time (*h*) and *ρ* is the material density (g cm^−3^). Four specimens were tested for each condition and the average values and relative standard deviations were calculated.

Additionally, insoluble degradation precipitates gathered after ethanol ultrasound cleaning of experimental specimens were centrifuged. The degradation products were then added to a solution of 50% ethanol and 50% nanopure water and vortexed, again centrifuged, dried and characterized by SEM-EDS and FTIR and XRD.

#### 2.3.1. Degradation characterization

The surface morphology, chemical compositions and phase composition of the degraded samples were examined by SEM, EDS, and XRD. The protective films formed on the samples were compared on the basis of their composition and average thickness. Additionally, sample surfaces were also analyzed by attenuated total reflectance Fourier transform infrared spectroscopy (ATR-FTIR, Agilent Cary 660 FTIR, Agilent Technologies, MN, USA) equipped with a deuterated l-alanine doped triglycine sulfate (DLa-TGS) detector and a Ge coated KBr beam splitter; the acquisition was performed in the wavelength range 4000–400 cm^−1^, with a resolution of 4 cm^−1^. Further, atomic emission spectroscopy (AES) was performed (model 4200 MP-AES, Agilent Technologies) to assess the release of Fe and Mg ions. A 10% nitric acid solution in deionized water was used to clean glassware for the digestion procedure, to avoid any kind of contamination.

## 3. Results

### 3.1. Microstructure of as-received specimens

The distribution of reinforcement varies with powder processing methods. In case of MM samples, Mg_2_Si particles were considerably refined and homogenously spread within the Fe matrix. As expected, this behavior was not seen in the sample prepared by conventional mixing.

**Fig. 1:**
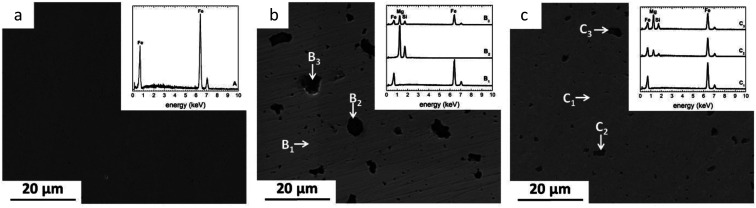
SEM micrographs of as-polished powder metallurgy Fe, Fe/Mg_2_Si composites and corresponding EDS spectra (a) PF, (b) MX, (c) MM.

The size distribution of Mg_2_Si was calculated using image analysis. The results showed the presence of coarse (∼12% in the range of 5–10 μm) and small (∼50% in the range of 1–4.9 μm and ∼38% in the range 0.3–0.99 μm) Mg_2_Si particles for MX samples. In contrast, high energy ball milling resulted in significantly decreased reinforcement size down to the sub-micrometer regime (Fig. 1c). For MM samples, (∼83% of reinforcement particles were in a range of 0.1–0.99 μm) and (17% particles exhibited size between 1–3 μm).

### 3.2. XRD and FTIR analysis of degraded samples

As the structure, chemical composition and mechanical properties of Mg_2_Si are different from those of pure Fe, the addition of reinforcement to the latter had an influence on the chemical composition and morphology of degradation products. Considering the degradation products on the surface samples, those formed on PF were different from those formed on composites.

**Fig. 2:**
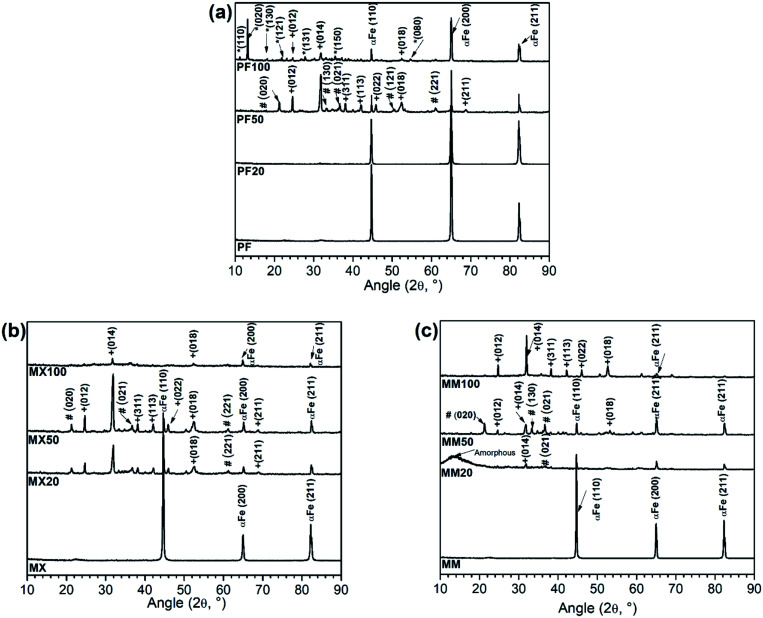
XRD patterns of (a) PF, (b) MX, (c) MM, in as received condition and after immersion test in modified Hanks' solution at different exposure intervals (20, 50 and 100 days, respectively). The identified phases are following: #, α-FeOOH (JCPDS #29-0713); +, FeCO_3_ (JCPDS #29-0696); *, Fe_3_^2+^(PO_4_)_2_·8H_2_O (JCPDS #30-0662).

XRD patterns of as-received and degraded Fe and Fe/Mg_2_Si composites are presented in Fig. 2.

**Fig. 3:**
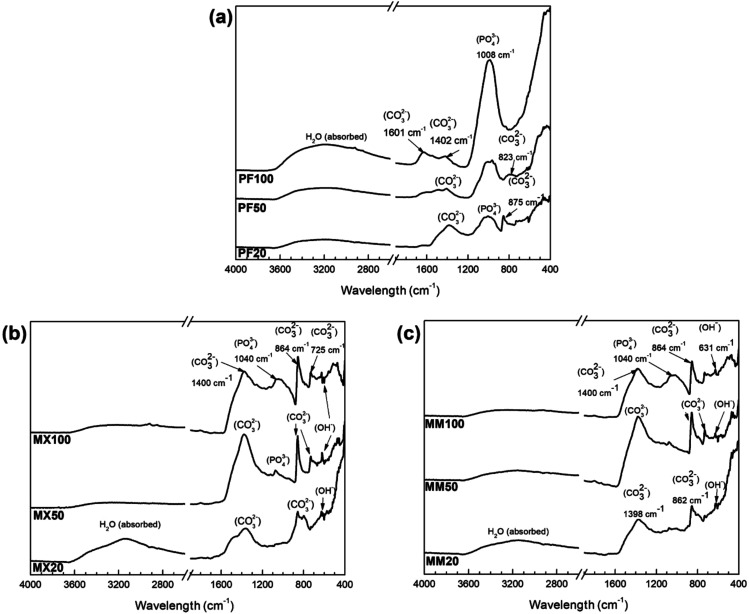
FTIR patterns of (a) PF, (b) MX, (c) MM samples after immersion test in modified Hanks' solution at different exposure intervals (20, 50 and 100 days, respectively).

**Fig. 4:**
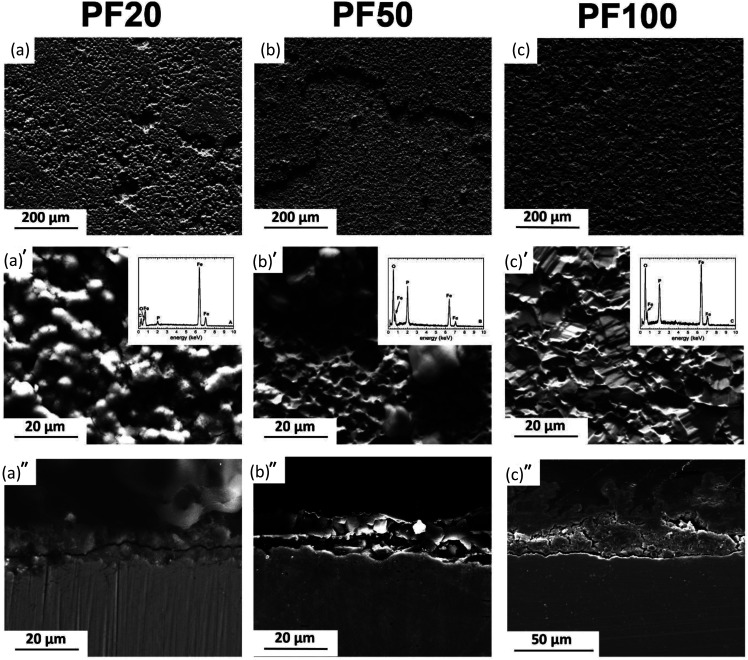
Typical degradation morphologies of PF samples at different exposure intervals to modified Hanks' solution: (a–a′) 20 days, (b–b′) 50 days and (c–c′) 100 days, and (a′′–c′′) corresponding cross-sections.

In case of PF20, XRD analysis showed only the presence of α-Fe (JCPDS #06-0696), as evidenced by the presence of (100), (200) and (211) peaks. Despite the formation of degradation products on the Fe surface, as evidenced by SEM-EDS studies (Fig. 4a–a′′), no reflections corresponding to phosphate compounds or iron oxides, hydroxides or carbonates were found. The presence of O and P, highlighted by EDS and FTIR (Fig. 3a and 6a) analyses, was due to the formation of insoluble phosphates so that this degradation layer could be amorphous or nanocrystalline, thus not detectable by XRD.

**Fig. 5:**
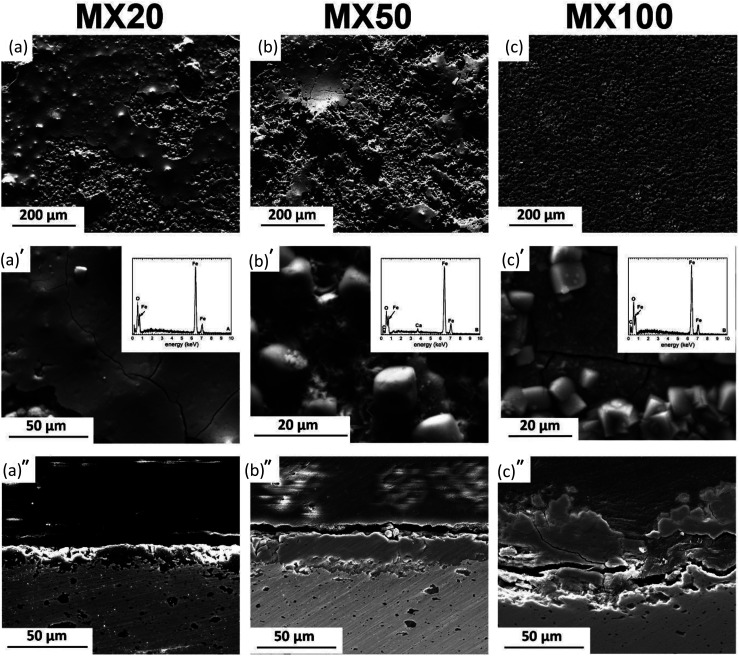
Typical degradation morphologies of MX samples at different exposure intervals to modified Hanks' solution: (a–a′) 20 days, (b–b′) 50 days and (c–c′) 100 days, and (a′′–c′′) corresponding cross-sections.

**Fig. 6:**
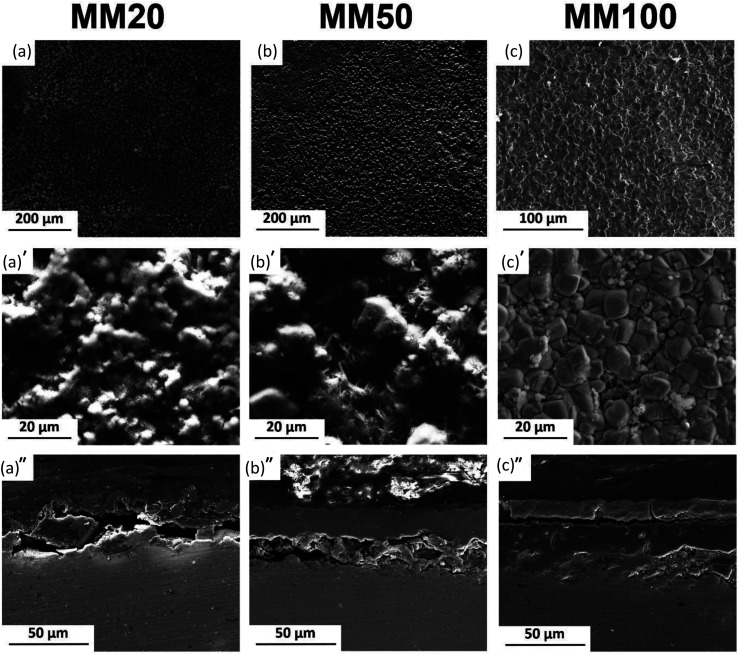
Typical degradation morphologies of MM samples at different exposure intervals to modified Hanks' solution: (a–a′) 20 days, (b–b′) 50 days and (c–c′) 100 days, and (a′′–c′′) corresponding cross-sections.

XRD spectra for PF50 revealed the presence of siderite (FeCO_3_-JCPDS #29-0696) and goethite (α-FeOOH-JCPDS #29-0713). The FeCO_3_ peaks corresponding to (012) and (014) planes were the most intense reflections. Other low-intensity siderite peaks corresponding to (113), (133), (022), (018) and (116) planes were identified. The presence of minor intensity peaks of goethite corresponding to the (020), (130), (021), (121) and (221) was also confirmed. α-Fe (110), (200) and (211) reflections exhibited reduced intensity compared to FP20; this fact can be attributed to the thickening of the protective film on the samples' surface with increasing exposure time.

PF100 XRD pattern showed the corresponding peaks to vivianite (Fe_3_^2+^(PO_4_)_2_·8H_2_O, JCPDS #30-0662) related peaks. The most intense reflections belonged to (020), (131) and (150) planes. Other lower intensity peaks such as (110), (130) and (121) are also present. In addition, peaks of FeCO_3_ were observed with the most intense reflections coherent with (012), (014) and (018) planes.

MX20 and MM20 XRD patterns (Fig. 2b and c) showed diffraction lines of Fe(iii) oxy-hydroxides and FeCO_3_. These peaks were more numerous and diffraction lines were more intense in case of MX type samples. MM showed only one peak of siderite (021), one peak of goethite (014) and α-Fe peaks corresponding to (100), (200) and (211) planes. Additionally, a poorly crystalline pattern showing an angle reflection around 10–15° has been observed for the composite prepared by mechanical milling, which could be attributed to the degradation layer of amorphous ferrous phosphates (Fig. 2c).^19^ Similarly, after 50 days of an experiment, the degraded surfaces of composites consist of siderite and goethite (Fig. 2b and c). The peaks of carbonate and hydroxide phases are more intense in case of Fe/Mg_2_Si prepared by mixing. The α-Fe peak intensity appears to decrease with increasing immersion time. It could be due to the growing protective layer formed on the surface, as it was further proved by SEM analyses (Fig. 5 and 6).

After 100 days immersion test, the diffraction pattern changed in a relevant way, suggesting the formation of a multilayered protective film on the surface of MX and MM samples. Fe(ii) carbonate peaks were found also for MX100 and MM100. In case of MX samples, only iron carbonate low-intensity peaks corresponding respectively to (104) and (018) planes were observed. MM showed several high-intensity peaks of FeCO_3_ belonging to (012), (014), (311), (113), (022), (018) planes. MX samples revealed two low-intensity peaks of α-Fe corresponding to (200) and (211) planes. For MM only one α-Fe peak was observed, coherent to (211) plane.

To determine the functional groups among different alloys after immersion testing, FTIR analysis was employed. Fig. 3 shows the sequential FTIR spectra of the surface of Fe and Fe/Mg_2_Si, which were exposed to the modified Hanks' solution at different intervals.

For pure Fe, the PO_4_^3−^ band is observed at 1026 cm^−1^, which is related to the asymmetric stretching of the ν3 group.^23^ Absorption intensity of this group increases with prolonged immersion time, which may indicate an improvement of the properties of the phosphate-based compound, making this phase more stable. This result is in good agreement with XRD (Fig. 2a) and SEM-EDS (Fig. 4) investigations, where at the end of the experiment crystalline vivianite was identified. Additionally, low-intensity CO_3_^2−^ absorption peaks vibration at around 1402, 875 and 825 cm^−1^ are visible. Further, the CO_3_^2−^ band at 1580–1620 cm^−1^ was detected on the Fe surface only after 100 days of experiment.^24^

The FTIR spectra of composites are similar, presenting the same pattern. They are dominated by a broad peak centered at around 1400 cm^−1^ and two sharp vibration peaks at around 860 and 725 cm^−1^.^25,26^ Furthermore, the results of FTIR indicate the formation of the phosphate functional group on the surface of the investigated composites only at the final stages of the corrosion experiment. The low-intensity absorption band in the range 1040–1140 cm^−1^ was observed on their surfaces after 50 and 100 days of the experiment.

Furthermore, at the final stages of the corrosion experiment, the formation of the phosphate functional group on the surface of the Fe/Mg_2_Si composites was revealed by FTIR analysis. In particular, the low-intensity absorption band in the range 1040–1140 cm^−1^ was observed on their surfaces after 50 and 100 days of the experiment. The broad peak at 3150 cm^−1^ with a shoulder at 3550 cm^−1^ can be assigned to OH^−^ stretching vibrations.^27^

### 3.3. Degradation morphologies of Fe and its composites after 20, 50 and 100 days of immersion in modified Hanks' solution

Fig. 4–6 show typical surface morphologies and corresponding cross sections for PF, and its composites (MM and MX) at 20, 50 and 100 days exposure intervals in modified Hanks' solution.

For PF20, the surface was covered with amorphous products containing Fe, O, P (Fig. 4a). Cross section revealed uniformly degraded surface with ∼15 μm thick deposit on the top.

As depicted in Fig. 4b, PF50 days revealed the surface covered by degradation products in the form of spherical particles adhering to the amorphous areas in the background, containing a high amount of P and O. The cross-sectional observation revealed a formation of a film with a uniform thickness of around 20 μm.

PF100 samples were covered entirely by phosphate. The cross-sectional analysis showed a characteristic double protective layer. This comprises the outer layer, mainly consisting of iron phosphate based compound alongside the inner layer mainly made of siderite and goethite (Fig. 4c). The total thickness varies from around 20 μm to 60 μm where the inner core uniformly covers the substrate and no pits are observed. The thickness of the outer layer varies significantly and can rise to 40 μm, while the thickness of the inner film varies from 10 to 20 μm.

MX and MM samples' surfaces (Fig. 5 and 6) showed the formation of a degradation layer with different features from PF samples. Nevertheless, both composites, prepared by mixing or mechanical milling exhibited similar surface features.

The surface of MX20 (Fig. 5a) was covered by a compact and shell-like structure containing Fe and O. Despite similar, Fe and O containing compounds found on the surface of MM20, their irregular and cotton balls morphology differs compared to those of composite prepared by mixing.

50 days of experiment resulted in nearly identical surface morphologies for both composites: sharp edges crystals were distributed uniformly on the areas visible in the background. The background surface contained mainly Fe, O, and traces of Ca. After cross-sectional observations, the protective layer on composites appears more robust than that of the pure Fe. Moreover, as seen in Fig. 5a′′–c′′, dissolution of the composite substrate is deeper compared to Fe with visible pits.

The protective layer thickness for all kinds of investigated specimens increased with exposure time. In case of the composites, the thickness of the deposit is greater than that measured for PF. Nevertheless, the solubility of final degradation products formed on the surface after 100 days of immersion differed significantly. PF was covered entirely by vivianite, while on the surface of Fe/Mg_2_Si composites siderite was detected. The degradation of Fe-based materials can develop homogenously on the surface or can be confined to specific sites featuring a pitting corrosion. More homogenous corrosion of PF and MM results in the development of uniform protective films (as seen in Fig. 4a′′–c′′ and 6a′′–c′′). Localized corrosion of MX produces mounds of degradation products (Fig. 5a′′–c′′).

The higher layer thickness for MM and MX is due to the occurrence of nonuniform galvanic corrosion between coarse reinforcement particles and Fe matrix. The increase in film thickness within 20 days of immersion in case of composites is greater than that of pure Fe and may be related to the susceptibility to the localized corrosion. The thinner film on the surface of Fe was related to the passivating property of the degraded sample surface.

### 3.4. Degradation precipitates

**Fig. 7:**
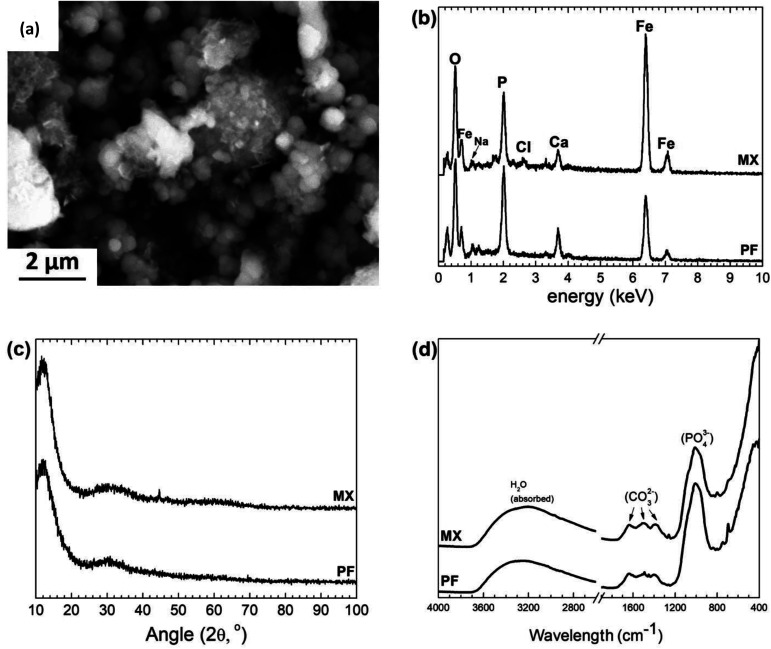
(a) Representative image of degradation products after 50 days of immersion of MX sample, (b) EDS, (c) XRD and (d) FTIR on degradation precipitates after 50 days of immersion.

The degradation precipitates were collected after ultrasound sample rinsing in ethanol solution. The representative morphology of the precipitates removed from the degraded MX sample after 50 days of immersion test is shown in Fig. 7a. The degradation products of PF and MX were essentially composed of a high amount of Fe, O, P, Ca. C and traces of Na, measured by EDS (Fig. 7b). This layer is in contact with the solution and is affected by its composition. As seen in XRD pattern (Fig. 7c) no crystalline phases associated with Fe compounds were detected. XRD showed only a presence of the broad maxima, which could be attributed to amorphous ferrous phosphate degradation layer. Fe oxide phases as lepidocrocite and goethite may be present here in addition to the other precipitates (such as phosphates and carbonates). FTIR (Fig. 7d) confirmed the presence of phosphates among the degradation products.

### 3.5. Degradation rates and ion release

**Fig. 8:**
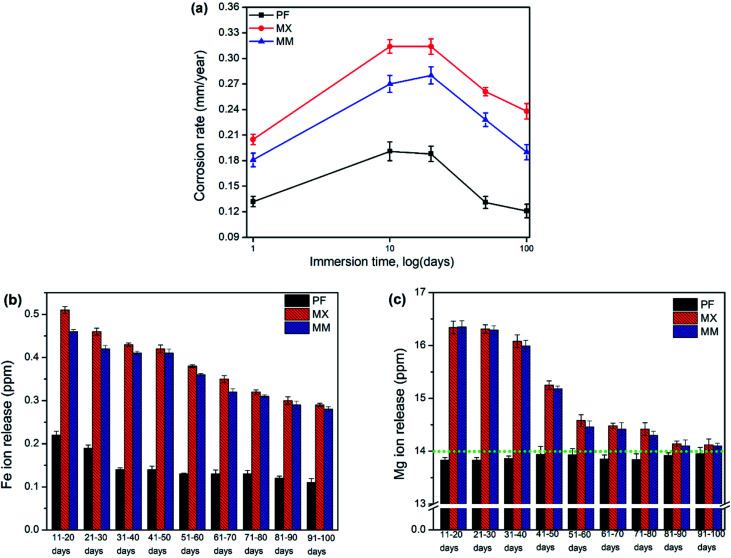
(a) Degradation rates of PF, MX, and MM after 1, 10, 20, 50 and 100 days of immersion (logarithmic scale), and ion release of (b) Fe and (c) Mg, after 20 to 100 days of static immersion test, *dotted line in figure. (c) Indicates the amount of Mg ions included in modified Hanks' solution ∼14 mg L^−1^.

Degradation rates of investigated materials were initially high, especially for low immersion durations and decreased rapidly as the protective film grew and provided increasing resistance to the corrosion reaction (Fig. 8a). AES analysis of exhausted solutions after immersion of Fe and its composites showed that Mg concentration was higher than Fe ions. It is important to notice that a low amount of Mg (∼14 mg L^−1^) is present in modified Hanks' solution (Fig. 8c).

The amount of ions in the exhausted solution after immersion test at longer intervals of exposure (50 and 100 days) revealed the decreasing concentration of Fe and Mg. Nevertheless, ion release in case of composites was always higher than that of PF. These results confirm explicitly the efficiency of Mg_2_Si in the increasing degradation rate of degradable Fe biomaterials, even at long-term exposures. During the corrosion process of metallic biomaterials in physiological solutions active and passive surfaces exist concurrently in contact with electrolyte.^28^ Therefore, the surface oxide/hydroxide/phosphate/carbonate film on the Fe and Fe/Mg_2_Si continuously pursues a process of partial dissolution and reprecipitation in modified Hanks' solution. When the dissolution rate is larger than that of reprecipitation, Fe and Mg ions are gradually released. When the metallic surfaces are covered with a thick and compact film, the ion release and degradation rates show a decreasing tendency. The composition of this protective film might change even though these precipitates are macroscopically stable.

## 4. Discussion

Body fluids are aqueous solutions consisting of inorganic and organic compounds and ions. Their combined effect is a key factor affecting the degradation of Fe-based alloys and composites.^29^ Hanks' modified solution is one of the most common simulated body fluids to perform *in vitro* degradation tests.^3,5,19^ The corrosion pattern of Fe and Fe/Mg_2_Si composites in this solution can be used to predict their *in vivo* behavior because its ionic concentration is similar to that in blood plasma.^22^ The degradation of Fe is a phenomenon triggered by surface conditions and it depends on the chemical composition of the corrosive medium, pH, temperature, and solubility of metallic ions released from the substrate.^19^

When Fe is immersed in a solution, it is oxidized to Fe^2+^ based on the following reaction:^30^

2 Fe → Fe^2+^ + 2e

Some of Fe^2+^ can be transformed to Fe^3+^ because of alkaline pH and the presence of oxygen in Hanks' solution; Fe(OH)_3_ is produced according to the following reactions:

3 1/2O_2_ + H_2_O + 2e^−^ → 2OH^−^

4 Fe^2+^ + 2OH^−^ → Fe(OH)_2_

5 Fe^2+^ → Fe^3+^ + e^−^

6 Fe^3+^ + 3OH^−^ → Fe(OH)_3_

**Fig. 9:**
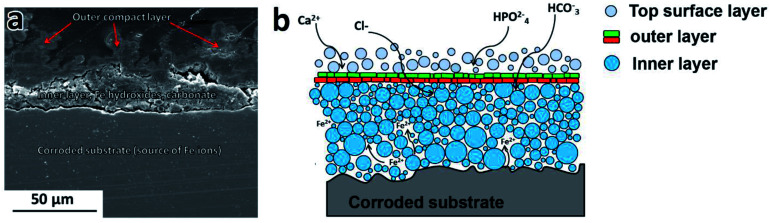
(a) SEM micrograph of PF100 cross-section showing the layered structure of protective films formed during degradation (b) schematic showing characteristic features of the degradation deposit.

Because of the presence of chloride ions, Fe(OH)_3_ is hydrolyzed and goethite (α-FeOOH) is formed. According to the Pourbaix diagram with increasing pH, the local H^+^ concentration at the metal-solution interface decreases, resulting in a formation of Fe oxides and hydroxides on the iron surface.^31^ Fe(OH)_2_ and Fe(OH)_3_ are solid phases with a low solubility (respectively *K*_sp_ (Fe(OH)_2_) = 8 × 10^−16^, *K*_sp_ (Fe(OH)_3_) = 4 × 10^−38^).^32^ The stability of these compounds is limited by the presence of carbonates/bicarbonates and phosphates, chloride ions and changes in the concentration of the degradation products.^33,34^ Subsequently, after immersion, apart from the contact with Cl^−^, HCO_3_^−^, HPO_4_^2−^, Fe and Fe/Mg_2_Si were also exposed to an environment rich in cations (such as Ca^2+^, Mg^2+^, Na^+^, K^+^*etc.*), dissolved oxygen and CO_2_. Therefore, in addition to the deposition of a Fe hydroxide layer, the presence of HCO_3_^−^, Cl^−^ and HPO_4_^2−^in modified Hanks' solution also promotes thermodynamically the precipitation of other phases. The corrosion deposit is thus reconstructed into layers of oxides, hydroxides, Fe and Ca carbonates and phosphates, as illustrated in the scheme in Fig. 9. Chloride, carbonate and sulfate anions, present in the body fluid are responsible for the formation of intermediate phases in the iron oxidation process. Fe_6_(OH)_12_CO_3_, Fe_4_(OH)_8_Cl and Fe_6_(OH)_12_SO_4_ complexes, known as green rust (GR), might be formed during Fe biodegradation.^35^ GR rust forms on the substrate surface in a pH range of 7 to 9 and has a substantial contribution to the degradation of Fe based biomaterials.^35^

The nature of the protective film (chemical composition and thickness) depends not only on the environmental parameters such as the buffer solution, pH, and temperature. Microstructural features (such as grain size, uniformity) and chemical composition of the substrate have a significant contribution to the corrosion susceptibility of Fe-based BMs.

The film formed on pure Fe in a slightly alkaline buffer solution containing phosphate species was reported to be composed of an inner layer of iron oxides and an outer part of iron phosphates.^36^ Other researchers reported that the presence of phosphates in the solution promotes Fe passivation, resulting in the formation of phosphates in either amorphous or crystalline form.^19,34,37^ It was reported by Refait *et al.*^37^ that the amorphous deposit created on the Fe surface was made of nanoparticles revealing a similar structure to that of carbonate-based green rust. The adsorption of phosphate species on these nanoparticles hindered their growth and transformation into a fully ripened Fe carbonates. It is well accepted that phosphate species show high affinity to Fe oxides and hydroxides.^32^ They can adsorb on their surfaces and then precipitate with dissolved Fe^2+^ species, therefore acting as an important precursor phase for vivianite formation. As evidenced by SEM-EDS analyses the formation of phosphate-based chaotically distributed compounds on the surface of Fe is observed at all stages of the corrosion experiment for PF samples. At the initial stages of immersion, Fe^2+^ ions react with phosphates present in Hanks' modified solution, resulting in amorphous phosphate precipitation. During the following stages of the degradation these compounds grew, forming finally protective, crystalline vivianite layer after 100 days (Fig. 2 and 4c). FTIR and XRD results confirmed coexistence of crystalline vivianite and siderite on PF samples surface after 100 days of immersion test. It was suggested that siderite having a higher solubility (*K*_sp_ = 5 × 10^−9^) protects growing vivianite from being attacked by corrosion. Higher concentration of Fe^2+^ ions, coming from siderite decomposition might favor the phosphate-based species formation.^38^ Further, the production of vivianite might be limited by lack of P ions in the solution and/or the amount of available Fe^2+^ ions.^38^

Degradation products found on the composites' surface vary compared to PF. As shown in Fig. 2, FeCO_3_ is formed on MX and MM surfaces more readily. Indeed, this could be due to increasing concentration of hydroxides coming from the dissolution of finely distributed reinforcement and thus, the local pH near to the surface raises followed by the formation of the iron carbonate layer.^5^ It was already found that pH is a dominant factor controlling the saturation state for siderite.^39,40^ Further, the deposition of iron carbonate can be promoted by environmental factors during an experiment. The 5% vol. CO_2_ atmosphere at pH = 7.4 leads to the formation of H_2_CO_3_. This dissociates and forms bicarbonate and hydrogen carbonate ions, contributing to the FeCO_3_ deposition. Additionally, modified Hanks' solution is a buffer of high capacity, which is in favor of FeCO_3_ formation.^41^

The degraded surfaces of MM50 samples are characterized by a denser and more homogeneous distribution of FeCO_3_ crystals (Fig. 6a′ and b′) compared to MX50 samples (Fig. 5a′ and b′). Finally, MM100 samples are entirely covered by a dense layer of siderite (Fig. 6c′). This difference can be ascribed to the initial microstructure of considered specimens. Markedly refined Mg_2_Si reinforcement particles were distributed homogenously through the Fe matrix (Fig. 1b and c). Reinforcement particles for MM samples might be responsible for a uniform rise of the local pH near the surface and therefore the formation of more homogenous and compact FeCO_3_ deposit. The proposed mechanism was supported by experimental investigation of corrosion initiation for Fe/Mg_2_Si composites prepared by different powder metallurgy methods.^5^

### 4.1. Protective film structure and formation

Stages of protective film formation involve the initiation of corrosion on the polished metallic surface. During this stage, oxidants existing in modified Hanks' solution reach a metal – solution interface and initiate corrosion process. Further, a growth of the protective deposit results in continued dissolution followed by a combination of degradation products oxidation and precipitation. With increasing thickness of the degradation layer, it becomes progressively difficult for the corrosive medium to diffuse to the metal–physiological solution interface. Different morphological features of the degradation layers start forming on the surfaces of PF, MX and MM samples at distinct stages of corrosion experiment as evidenced by SEM observations (Fig. 4–6). The change in the ratio of the inner and outer layer, as can be seen in Fig. 4–6a′′ and b′′, indicates changes in the transport of ionic species. This depends on the composition of the substrate as well as the size and distribution of reinforcement in case of MM and MX composites. We observed that uniform corrosion results in the development of more uniform films (PF, MM in Fig. 4a′′–c′′ and 6a′′–c′′, respectively), while localized corrosion can produce mounds of corrosion products (as in case of MX samples – Fig. 5 a′′–c′′).

With increasing exposure time the reaction mechanism for carbonates/phosphates and hydroxides is significantly slower and mutual diffusion of the elements from the substrate and Hanks' modified solution is reduced. Principal diffusion pathways are provided by the capillaries in the porous corrosion layers where Fe ions diffuse from the bulk material toward the solution/surface interface, whereas ions present in the body fluid diffuse in the opposite direction (as depicted in Fig. 9b). Thus, the degradation rates are reduced with degradation deposit thickening and its densification. For example, the compact outer phosphate-based layer (on the surface of PF100) could hinder ion diffusion and further oxidation at the metal–layer interface. Fe-based phosphates are in fact characterized by very low solubility.^42^

The chemical composition, the stability, and the sequence of protective film formation on the surface depend on the microstructure and composition of the substrate as well as the fabrication method. The characteristic features of these deposits proposed based on the detailed investigations of degraded samples included: (i) degraded substrate (PF, MX or MM sample surface), (ii) inner layer, (iii) outer layer, and (iv) top surface layer as seen in Fig. 9.

The degraded metal surface underneath a protective film is a main source of the Fe ions. After the protective film has formed, further substrate dissolution proceeds at a slower rate, resulting in the continued growth of the degradation products film.

The inner protective film siderite, goethite, and lepidocrocite were identified by XRD as components of the inner layer. This region localized closer to the metal surface is expected to contain a higher amount of Fe. EDS analyses showed increased Fe and O concentration in this part of the deposit.

The outer layer covers the inner one. SEM images (Fig. 4–6a′′ and b′′) of the cross-section of PF, MX and MM show the compact layer of varying thickness (20 to 80 μm), depending on immersion time and composition of the substrate. This layer acts as a barrier between modified Hanks' solution, Fe ions and solids present inside of the inner layer.

The top surface layer consists of loosely held particles on the top of the outer layer made of Fe oxides, hydroxides in addition to the precipitates of carbonates and phosphates (as seen in Fig. 7).

## 5. Conclusions

In this article, the *in vitro* degradation properties of pure Fe and Fe/Mg_2_Si composites prepared by different PM techniques were investigated to evaluate their potential as a biodegradable implant material. Their degradation performance was investigated *via* static immersion test in modified Hanks' solution at different exposure intervals up to 100 days. The degradation mechanism in physiological solution was proposed, and degradation rates (obtained from the immersion tests) were discussed. At subsequent stages of degradation, the structure and chemical composition of the formed protective layers had an important impact on the dissolution of the experimental specimens. Further, the results revealed the role of Mg_2_Si in the composition and stability of the protective films formed during the static corrosion experiment.

Fe/Mg_2_Si showed higher degradation rates than that of pure iron at all stages of the degradation experiment (*i.e.* 0.12 *vs.* 0.24 mm per year for PF100 and MX100 samples, respectively). Indeed, the degradation of pure Fe is mainly related to the properties of Fe itself, while in Fe/Mg_2_Si composites it depends on the distribution and size of the reinforcement particles. Thus, the features of the degradation products obtained after 100 days of the experiment varied with the substrate chemical composition and its microstructure. Corroded surfaces of pure iron samples were entirely covered by stable iron phosphate. In contrast, Fe/Mg_2_Si composites exhibited the presence of carbonates at the latest stages of the test. A shift of the corrosion regime from localized to a more uniform was observed in case of the mechanically milled composite, mainly due to reinforcement refinement.

This study provided a basis for the processes of protective film formation in modified Hanks' solution, enabling detailed identification of their characteristic features. In particular, the ability to tune long-term degradation behavior of the composites as a function of reinforcement properties and manufacturing method was experimentally verified.

## Conflicts of interest

There is no conflict to declare.

## Supplementary Material
